# Insulin Sensitivity and Testicular Function in a Cohort of Adult Males Suspected of Being Insulin-Resistant

**DOI:** 10.3389/fmed.2018.00190

**Published:** 2018-06-26

**Authors:** Patricio H. Contreras, Felipe G. Serrano, Ana M. Salgado, Pilar Vigil

**Affiliations:** ^1^Reproductive Endocrinology Department, Reproductive Health Research Institute, Santiago, Chile; ^2^Fundación Médica San Cristóbal, Santiago, Chile; ^3^Vicerrectoría de Comunicaciones, Pontificia Universidad Católica de Chile, Santiago, Chile

**Keywords:** insulin resistance, male hypogonadism, waist circumference, pancreatic suppression test, ROC analysis

## Abstract

A cohort of 141 males (18–80 yo, 42.9 ± 12.9) strongly suspected of being Insulin Resistant (IR) was prospectively studied by determining their insulin sensitivity (Pancreatic Suppression Test, PST) and testicular function (total testosterone and SHBG). The subjects were labeled as IR when the Steady State Plasma Glucose (SSPG) was ≥150 mg/dL and Non-Insulin Resistant (NIR) when SSPG was <150 mg/dl; similarly, the subjects were labeled as Hypogonadal (HYPOG) when total testosterone was ≤3.0 ng/mL and Eugonadal (EUG) when total testosterone was >3.0 ng/mL. Two out of three subjects turned out to be IR, while around one in four subjects were HYPOG. Contingency analysis indicated a significant interdependence between insulin resistance and hypogonadism (chi-square was 4.69, *p* = 0.0303). Age (>43 yo) predicted hypogonadism (AUROC 0.606, *p* = 0.0308). Twice as many HYPOG subjects were IR as compared with EUG subjects. Also, HYPOG subjects exhibited higher SSPG values as compared with EUG subjects. Statistically, neither Weight nor BMI predicted hypogonadism, while Waist Circumference (>110 cm) was only a mediocre predictor (AUROC 0.640, *p* = 0.009). SSPG (>224 mg/dL) on the other hand, was the best predictor of hypogonadism (AUROC 0.709, *p* = 0.002), outperforming Waist Circumference (half of the subjects with an SSPG >224 mg/dL were HYPOG). Age did not predict insulin resistance, while Weight (>99 kg), BMI (>29), and especially, Waist Circumference (>99 cm, AUROC 0.812, *p* < 0.0001) were all predictors of insulin resistance. Almost 90% of the subjects with a waist circumference >99 cm was IR. As a logical consequence of the selection criteria (various clues suggesting insulin resistance), most subjects with normal weight in this cohort were IR (53.3%) while 20% were HYPOG. On the other hand, 13.6% of the obese subjects were NIR, and 2 out of 3 of them were both NIR and EUG. In conclusion, Waist Circumference predicted both insulin resistance (>99 cm) and hypogonadism (>110 cm), suggesting that the first hit of abdominal obesity is insulin resistance and the second hit is male hypogonadism. Normal weight did not protect from IR, while a relevant proportion of obese subjects were NIR (with 2/3 being also EUG).

## Introduction

To summarize the intricate current knowledge linking insulin resistance and hypogonadism in males, it is convenient to put it together in a temporal sequence, spanning almost 3 decades of research. In 1990, Zumoff et al. showed that serum levels of free testosterone were reduced in obese men in proportion to their obesity (1). Thereafter, the Telecom Study, published in 1992, disclosed an inverse relationship between serum levels of testosterone and insulin in over a thousand healthy adult males between 20 and 60 years of age (2). The interrelationships between insulin resistance, body fat distribution and sex hormones in men were delineated by Haffner et al. (3). Summarizing their results, higher waist/hip ratios and lower testosterone values were strongly associated with a decrease in total and non-oxidative glucose disposal in a group of 87 males. In 1996, Haffner et al. reported that low levels of testosterone and SHBG predicted the development of type 2 diabetes in men (4). In 2005, Pitteloud et al. showed that the secretory capacity of the Leydig cell was reduced in insulin-resistant male subjects (5). In fact, insulin sensitivity, as measured by the euglycemic hyperinsulinemic clamp, correlated positively with basal serum testosterone and with testosterone secretion in response to hCG, when the male subjects were rendered hypogonadal with GnRH antagonists. Conversely, Pitteloud et al. in 2005 suggested that low testosterone levels may further deteriorate insulin sensitivity in insulin-resistant males by impairing key mitochondrial functions (6), implying a bi-directional, opposing relationship between insulin sensitivity and testicular malfunctioning.

A key new knowledge was reported in 2006 by Kapoor et al. testosterone replacement in hypogonadal males with type 2 diabetes improved insulin resistance, glycemic control, visceral adiposity, and cholesterol levels (7). This was relevant since only 2 years before, Dhindsa et al. reported that male hypogonadism is a frequent finding among type 2 diabetes patients (8).

On the other hand, the HIM Study in 2006 (9) suggested that abdominal obesity in males could be the most frequent cause of acquired hypogonadism. In fact, more than half of the obese males (52.4%) had serum testosterone levels below 3 ng/mL, a figure higher than the corresponding figure of diabetic subjects (50%) in the same study. Similarly, waist circumference in males was clearly associated with the risks of insulin resistance and diabetes, in a study published by Mamtani et al. (10).

Abdominal obesity, therefore, seems to be a common link between insulin resistance and male hypogonadism. Male hypogonadism has bi-directional, positive relationships with both abdominal obesity and insulin resistance. Consequently, a substantial weight loss in obese males is associated with a reversion of their hypogonadism, as shown by Corona et al. (11). Conversely, in 2016 Saad et al. (12) have shown that testosterone supplementation in hypogonadal obese males is a potential, promising therapy for obesity itself.

While male hypogonadism is widely accepted as causing insulin sensitivity impairment, testosterone treatment is not uniformly considered as an insulin-sensitizing maneuver. For instance, in 2016, Dhindsa et al. published a randomized clinical trial (13) with 44 hypogonadal diabetic males who received intramuscular testosterone (250 mg every 2 weeks for 24 weeks) or placebo. The glucose infusion rate (euglycemic clamp) rose by 32% in those treated with testosterone, but it did not change in those treated with placebo. In addition, the expression of insulin-signaling genes in adipose tissue was reduced in hypogonadal subjects and was upregulated following testosterone treatment. Also, there was a reduction in subcutaneous fat and an increase in lean body mass in those treated with testosterone. Finally, testosterone treatment reduced the circulating levels of free fatty acids, C reactive protein, interleukin-1β, tumor necrosis α, and leptin. The same year though, Magnussen et al. found, in a randomized controlled trial that testosterone supplementation given to hypogonadal male diabetes did not improve insulin sensitivity tested with euglycemic clamp (14).

Moreover, the insulin-sensitizing ability of exogenous testosterone in eugonadal, non-insulin-resistant male subjects has never been shown. In fact, in 2018 Huang et al. (15), reported that 3 years of testosterone supplementation in eugonadal or mildly hypogonadal older men did not result in an improvement in insulin sensitivity.

While the mechanisms explaining a reduced insulin sensitivity in hypogonadal men are partially clear (6), the mechanisms explaining the appearance of male hypogonadism in insulin-resistant subjects are obscure. It is even likely that a condition closely related to insulin resistance, such as hyperleptinemia (leptin resistance), rather than insulin resistance *per se*, is causing testicular malfunctioning (16), as suggested by Isidori et al. in 1999.

Despite all the conquered new knowledge, linking male hypogonadism with obesity and insulin resistance, the puzzle is still incomplete and several pieces of information seem to be missing. For instance, the prevalence and magnitude of hypogonadism among unselected adult insulin-resistant males, as well as its quantitative association with diminished insulin sensitivity have not been reported to our knowledge. To address this issue, we analyzed a cohort of adult males highly suspicious of being insulin-resistant in whom we measured insulin sensitivity directly, and simultaneously determined their gonadal status. As shown below, our results suggest that a relatively mild hypogonadism is a frequent co-morbidity in insulin-resistant males.

## Materials and methods

We prospectively studied a cohort of 141 consecutive unselected males strongly suspected of being insulin-resistant (IR) on clinical grounds (personal and family history, suggestive lipid profile—elevated triglycerides plus reduced HDL levels—, hyperuricemia, and/or suspicious physical findings, such as abdominal obesity, acanthosis nigricans, achrocordons, or hypertension, as well as subjects combining two or more suggestive clues). Those with known diabetes mellitus or using potentially interfering medications (such as glucocorticoids, beta-blockers, thiazides, statins, antipsychotics, protease inhibitors, opioids, or metformin) were excluded. Weight and height were obtained and body mass index (BMI) was calculated (weight in kilograms/[height in meters squared]). This research was approved by the Ethics Committee of the Reproductive Health Research Institute (RHRI).

Waist circumference was measured in centimeters by a single observer at the narrowest waist, as described by Lean et al. (17). Following an overnight fast, blood was drawn to measure total testosterone and SHBG. Testosterone was measured with a radioimmunoassay kit provided by Dia-Source Immunoassays, Belgium. SHBG was measured with DSL's immuno-reactive kits. Free testosterone was calculated with the equations first described by Södergard (18) and later by Vermeulen (19).

### Pancreatic suppression test (20–22)

Testing was done after a 12-h fasting. The PST involves the continuous infusion of glucose (267 mg/m^2^/min), crystalline insulin (32 mU/m^2^/min) and octreotide (0.27 mcg/m^2^/min, to suppress endogenous insulin production) for 3 h. Under such conditions, endogenous insulin production is suppressed by octreotide and the steady-state serum insulin is raised in the subjects to stimulate muscle uptake of glucose. During the PST serum insulin levels are kept stable (octreotide suppresses its pancreatic secretion and exogenous crystalline is infused according to the body's surface), while allowing serum glucose to change, as a function of muscle insulin sensitivity. The steady-state-plasma glucose (SSPG, the averaged 4 final glucose values: 150-160-170-180 min) becomes then an inverse function of insulin sensitivity. Plasma glucose measurements were obtained at 0-30-60-90-120-150-160-170 and 180 min. SSPG values <150 mg/dL indicated a non-insulin-resistant (NIR) status whereas values of ≥150 mg/dL indicated an IR status. Additionally, each patient was categorized as eugonadal (EUG, total testosterone >3.0 ng/mL) or hypogonadal (HYPOG, total testosterone ≤3 ng/mL). The sample to measure serum testosterone was taken at the start of the PST.

Plasma glucose was measured with the glucose-oxidase method. Results are expressed as the mean ± standard error of the mean (SEM).

### Contingency analysis

We used Chi-square analysis to study the possible interdependence between high SSPG and low testosterone levels, having categorized each subject as IR or NIR and additionally, as HYPOG or EUG. Statistical significance was established with a *p* < 0.05.

### Bayesian analysis

By using True Positive (TP), False Negative (FN), False Positive (FP), and True Negative (TN) numbers we computed sensitivity [TP/(TP+FN)] and specificity [TN/(TN+FP)]. Youden index, the best indicator of the predicting power of a predictor, was equal to (sensitivity+specificity-1). A perfect test will have neither FN nor FP results, so both sensitivity and specificity will be equal to 1 and the Youden index will reach a maximal value, equal to 1.

### ROC analysis

ROC analysis for both elevated SSPG (IR) and low testosterone (HYPOG) was computed with the help of the XLSTAT package (https://www.xlstat.com) to identify the optimal cut-offs of their potential predictors. A graph is constructed by the program plotting sensitivity (true positive rate, y-axis) against the false positive rate (1-specificity, x-axis). The program provides the area under the ROC curve (AUROC), and this area is compared with the null hypothesis (AUROC not different than 0.5). A useless test has an AUROC of 0.5, whereas a perfect test has an AUROC of 1. The Youden's Index equals zero with an AUROC of 0.5 whereas it equals 1 with an AUROC of 1.

An AUROC ≥0.9 indicates an outstanding discrimination; an AUROC ≥0.8 and <0.9 indicates an excellent discrimination, whereas an AUROC ≥0.7 and <0.8 indicates an acceptable discrimination. An AUROC <0.7 indicates an increasingly poorer discrimination (23). When the AUROC reaches 0.5 the test is unable to discriminate at all.

The optimal cut-off value for a given predictor found by ROC analysis is the one associated with the highest sum of sensitivity and specificity, and therefore, with the highest Youden's value.

## Results

The ages of the male subjects in this cohort ranged from 18 to 80 years (42.9 ± 12.9). Considering the whole cohort, 37/141 subjects were HYPOG (26.24%, around 1 in 4) and 94/141 subjects were IR (66.66%, 1 in 3). In this cohort, 15 subjects (10.6%) had a normal BMI, 60 subjects (42.6%) were overweight, and the remainder 66 subjects (46.8%) were obese. Their respective BMIs were 23.8 ± 0.3, 27.6 ± 0.2, and 33.2 ± 0.4 Kg/m^2^ while their SSPG values were 148.6 ± 18.2, 164.5 ± 9.7, and 229.9 ± 9.4 mg/dL, respectively.

IR and HYPOG were both found in varying proportions, in the three groups of BMI. Among normal-weight subjects 8/15 (53.3%) were IR and 3/15 (20%) were HYPOG; among overweight subjects, 29/60 (48.3%) were IR and 14/60 (23.3%) were HYPOG and, finally, among obese subjects 57/66 (86.4%) were IR and 20/66 (30.3%) were HYPOG. Total testosterone values (mean ± SEM) followed an inverse tendency with increasing BMIs: 5.2 ± 0.6, 4.2 ± 0.2, and 4.0 ± 0.2 ng/mL. To focus on abdominal obesity, we arranged these subjects into 3 tertiles of waist circumference: 90.6 ± 0.6, 101.2 ± 0.5, and 114.1 ± 1.0 cm. Again, SSPG values (mean ± SEM) rose with increasing waist circumferences: 152.6 ± 11.4, 177.5 ± 10.1, and 251.7 ± 9.7 mg/dL. Similarly, total testosterone values diminished with increasing waist circumferences: 4.6 ± 0.3, 4.4 ± 0.2, and 3.6 ± 0.2 ng/mL.

When the subjects were arranged into 3 tertiles of SSPG values (101 ± 3.9, 190.6 ± 3.3, and 290.7 ± 5.3 mg/dL), mean ± SEM total testosterone values fell in the highest tertile of SSPG values: 4.5 ± 0.2, 4.6 ± 0.3, and 3.5 ± 0.2 ng/mL.

When the subjects were arranged into 3 tertiles of total testosterone values (2.6 ± 0.1, 4.0 ± 0.1, and 6.2 ± 0.2 ng/mL) the corresponding values of waist circumference diminished accordingly: 106.2 ± 1.7, 100.7 ± 1.3, and 98.7 ± 1.5 cm; SSPG values also diminished proportionally with each tertile of increasing testosterone values: 228.2 ± 13.2, 178.1 ± 10.5, and 173.9 ± 10.8 mg/dL.

Table 1 shows that 64 subjects (45.39%) were both IR and EUG, 40 subjects (28.37%) were both NIR and EUG, 30 subjects (21.28%) were both IR and HYPOG and only 7 subjects (4.96%) were both NIR and HYPOG. 81.08% of the HYPOG subjects were also IR.

**Table 1:** Classification of the cohort according to insulin sensitivity and gonadal status.

**NIR and Eugonadal**	**NIR and Hypogonadal**
*n* = 40	*n* = 7
28.37%	4.96%
**IR and Eugonadal**	**IR and Hypogonadal**
*n* = 64	*n* = 30
45.39%	21.28%

*Only 40/141 (28.37%) subjects were neither IR nor HYPOG in the cohort; the remainder 101 subjects (71.63%) had either IR alone (64/141 = 45.39%), HYPOG alone (7/141 = 4.96%), or IR plus HYPOG (30/141 = 21.28%). Note that 30/37 (81.08%) of the HYPOG subjects were also IR*.

Table 2 shows that being IR increased the relative risk (RR) of being HYPOG: RR = 31.91/14.89 = 2.143.

**Table 2:** Gonadal status according to insulin sensitivity.

**Insulin sensitivity**	**Eugonadism**	**Hypogonadism**
**NIR**		
*n* = 47 (33.3%)	40/47 (85.11%)	7/47 (14.89%)
**IR**		
*n* = 94 (66.6%)	64/94 (68.09%)	30/94 (31.91%)

*In this cohort, being IR increased the relative risk of being HYPOG: 31.91/14.89 = 2.143*.

Table 3 shows that being HYPOG modestly increased the relative risk RR of being IR: RR = 81.08/64.41 = 1.259.

**Table 3:** Prevalence of insulin resistance according to gonadal status.

**Gonadal status**	**IR present**	**IR absent**
**Eugonadal**		
*n* = 104 (73.76%)	67/104 (64.42%)	37/104 (35.58%)
**Hypogonadal**		
*n* = 37 (26.24%)	30/37 (81.08%)	7/37 (18.92%)

*In this cohort, being HYPOG modestly increased the relative risk of being IR: 81.08/64.41 = 1.259*.

Table 4 shows the characteristics of the cohort: the majority (66.6%) was IR and the remainder 33.3% was NIR, whereas only a minority of the subjects (26.24%) was HYPOG. While age and height were not different in IR as compared with NIR subjects, weight, BMI, waist circumference and waist/height ratios were statistically higher in IR subjects. While height was not different in EUG compared with HYPOG subjects, the higher weight in HYPOG subjects as compared with EUG subjects fell short of statistical significance (*p* = 0.053). On the other hand, age, BMI, waist circumference, and waist/height ratios were statistically higher in HYPOG as compared with EUG subjects.

**Table 4:** Characteristics of the studied male population (*n* = 141).

**Variable**	**IR**	**NIR**	***p*-value**	**HYPOG**	**EUG**	***p*-value**
	***n* = 94 (66.66%)**	***n* = 47 (33.33%)**		***n* = 37 (26.24%)**	***n* = 104 (73.76%)**	
Age (years)	43.4 ± 1.3	41.7 ± 1.9	0.459 NS	46.2 ± 1.9	41.7 ± 1.3	0.049
Weight (Kg)	93.2 ± 1.7	83.3 ± 1.5	<0.0001	94.5 ± 2.9	88.2 ± 1.5	0.053 NS
Height (m)	1.74 ± 0.01	1.73 ± 0.01	0.220 NS	1.74 ± 0.01	1.74 ± 0.05	0.999 NS
BMI (Kg/m^2^)	31.0 ± 0.4	27.5 ± 0.4	<0.0001	31.5 ± 0.8	29.2 ± 0.3	0.019
Waist circumference (cm)	105.6 ± 1.1	94.5 ± 1.0	<0.0001	107.0 ± 2.0	100.1 ± 1.0	0.034
Waist/height ratio	0.606 ± 0.01	0.547 ± 0.01	<0.0001	0.615 ± 0.01	0.576 ± 0.01	0.0025

*Age was not different in IR compared with NIR subjects, while it was higher in HYPOG compared with EUG subjects; Weight was higher in IR compared with NIR subjects, while the higher value of Weight in HYPOG subjects compared with EUG subjects fell short of statistical significance; Height was not different in either group; BMI differences, when IR and NIR subjects were compared, were more pronounced than they were when HYPOG and EUG subjects were compared. Waist Circumference differences were more pronounced when IR and NIR subjects were compared than they were when HYPOG and EUG subjects were compared. The same phenomenon was observed when Waist/Height ratios were examined in both comparisons*.

Table 5 describes the gonadal status and the SSPG values of the studied population. Total testosterone, SHBG, and calculated free testosterone were not different when IR and NIR subjects were compared; in contrast, SSPG values, by design, were much higher in IR as compared with NIR subjects. When HYPOG and EUG patients were compared total testosterone and calculated free testosterone were, as expected by design, much lower in HYPOG subjects. While SHBG levels were lower in HYPOG subjects as compared with EUG patients, this difference did not reach statistical significance. SSPG values were higher in HYPOG patients when compared with EUG subjects.

**Table 5:** Gonadal status and SSPG values of the studied population.

**Variable**	**IR**	**NIR**	***p*-value**	**HYPOG**	**EUG**	***p*-value**
	***n* = 94**	***n* = 47**		***n* = 37**	***n* = 104**	
Total						
Testosterone (ng/mL)	4.1 ± 0.2	4.5 ± 0.2	0.177 NS	2.4 ± 0.1	4.9 ± 0.2	<0.0001
SHBG (nMol/L)	26.5 ± 2.1	29.7 ± 1.8	0.183 NS	24.5 ± 1.6	28.7 ± 1.4	0.052 NS
Calculated Free						
Testosterone (pg/mL)	98.4 ± 7.2	102.4 ± 6.3	0.626 NS	57.3 ± 2.6	114.8 ± 4.5	<0.0001
SSPG (mg/dL)	239.6 ± 8.6	101.0 ± 3.9	<0.0001	243.1 ± 14.7	175.7 ± 7.1	<0.001

*Total testosterone, SHBG and calculated free testosterone were statistically not different in IR and NIR subjects, while SSPG values, by design, were much higher in IR subjects compared with NIR males; total testosterone, SHBG and calculated free testosterone were, by design, much lower in HYPOG subjects compared with EUG males and, as suspected, SSPG values were higher in HYPOG males than in EUG subjects*.

To find out whether insulin resistance and hypogonadism had a statistically significant interdependence between them we ran a contingency analysis, as shown in Table 6. Chi-Square was 4.688, with 1 degree of freedom and the *p*-value was 0.0303 (<0.005), indicating a significant interdependence between insulin resistance and hypogonadism in this cohort.

**Table 6:** Contingency analysis table.

	**IR Subjects *n* = 94 (0.6666)**	**NIR Subjects *n* = 47 (0.3333)**
**Hypogonadal subjects**		
*n* = 37 (0.2624)	Observed = 30 Expected = 94^*^0.2624 = 24.67	Observed = 7 Expected = 47^*^0.2624 = 12.33
**Eugonadal subjects**		
*n* = 104 (0.7376)	Observed = 64 Expected = 94^*^0.7376 = 69.33	Observed = 40 Expected = 47^*^0.7376 = 34.67

*Chi-Square = 4.688, 1 degree of freedom, p = 0.003 (<0.05), indicating a significant interdependence between insulin resistance and hypogonadism in this cohort*.

The correlation matrix between the variables is shown in Table 7. SSPG values were positively more strongly correlated (*p* < 0.05) with waist circumference (abdominal obesity) with a correlation coefficient of 0.511, than with BMI (generalized obesity), with a correlation coefficient of 0.434. SSPG was also negatively correlated with total testosterone (*r* = −0.241) and calculated free testosterone (*r* = −0.169). Surprisingly, although SSPG was correlated negatively with SHBG, the correlation coefficient (*r* = −0.107) did not reach statistical significance.

**Table 7:** Correlation matrix between variables.

Weight	**1**								
Height	**0.402**	**1**							
BMI	**0.708**	0.027	**1**						
Age	0.129	0.065	−0.128	**1**					
Waist	**0.734**	0.156	**0.875**	0.058	**1**				
SSPG	**0.314**	0.117	**0.434**	0.147	**0.511**	**1**			
Testosterone	0.090	**0.209**	**−0.276**	**−0.226**	**−0.335**	**−0.241**	**1**		
Calculated Free T	0.012	**0.198**	−0.131	**−0.481**	**−0.230**	**−0.169**	**0.849**	**1**	
SHBG	**0.192**	0.004	**−0.269**	**0.491**	**−0.216**	−0.107	**0.258**	**−0.252**	**1**
	Weight	Height	BMI	Age	Waist	SSPG	Testosterone	Calculated Free T	SHBG

*Correlation figures in bold case are significant with a p < 0.05. SSPG values correlated positively better with WC values, compared with BMI or weight values. Testosterone values correlated negatively better with WC values, compared with BMI or age values*.

Testosterone values were negatively correlated more strongly with waist circumference (*r* = −0.335) than with BMI (*r* = −0.276). Testosterone values were also significantly and negatively correlated with age (*r* = −0.226), and positively correlated with SHBG (*r* = 0.258) and, unexpectedly, with height (*r* = 0.209).

Waist circumference (WC) was, as expected, highly correlated with BMI (*r* = 0.875). The regression equation between these two parameters of adiposity was: BMI = −4.0395 + 0.3323^*^WC (therefore, predicted BMI is approximately, one-third of the WC minus 4). By applying the above equation, a WC of 99 cm corresponded in this cohort, to a BMI of 28.9 and a WC of 110 cm corresponded to a BMI of 32.5.

WC was also negatively correlated with total testosterone (*r* = −0.335), calculated free testosterone (*r* = −0.230), and SHBG (*r* = −0.216). So, SHBG was negatively correlated with waist circumference but not with SSPG. As already stated, WC was positively and strongly correlated with SSPG (*r* = 0.511).

Of note, age was negatively correlated more strongly with calculated free testosterone (*r* = −0.481) than with total testosterone (*r* = −0.226). This is attributable to the strong positive correlation between age and SHBG (*r* = 0.491).

Table 8 shows the significant predictors of elevated SSPG (IR): the strongest predictor of SSPG was WC (AUROC = 0.812, sensitivity = 0.809, specificity = 0.755, Youden index = 0.564), followed by waist/height ratio (AUROC = 0.789, sensitivity = 0.713, specificity = 0.809, Youden index = 0.521). Despite that weight had a better AUROC than BMI (0.772 vs. = 0.760), the Youden index of BMI was higher than the respective value of weight (0.468 vs. 0.404). The cut-offs for these predictors were: WC >99 cm, BMI >29 Kg/m^2^, weight >91 Kg and waist/height ratio >0.575. It must be stressed though that a WC ≤99 cm did not protect against IR: in fact, despite that none of the normal weight subjects had a WC >99 cm, the majority of the subjects (8/15) were IR.

**Table 8:** Significant predictors of elevated SSPG (insulin resistance).

	**Weight (Kg)**	**BMI (Kg/m^2^)**	**Waist/Height**	**Waist (cm)**
AUROC	0.772	0.760	0.789	0.812
*p*-value	<0.0001	<0.0001	<0.0001	<0.0001
Cut-off value	>91	>29	>0.575	>99
Sensitivity	0.787	0.766	0.713	0.809
Specificity	0.617	0.702	0.809	0.755
Youden Index	0.404	0.468	0.521	0.564

*Waist outperformed (excellent discrimination) Waist/Height, BMI, and Weight (acceptable discrimination) at predicting SSPG values. Hypogonadism did not predict insulin resistance*.

To see the usefulness of a WC >99 cm at identifying IR subjects, we computed the mean ± SEM SSPG value in subjects with a waist circumference ≤99 cm and compared it with the mean ± SEM SSPG value found in subjects with a waist circumference >99 cm: 142.8 ± 9 (*n* = 64) vs. 231.9±8.1 (*n* = 77; *p* = 2.9^−10^), a highly significant difference. Moreover, 78 subjects of the cohort had a WC >99 cm; of them, 89.74% were IR and the remainder 10.26% were NIR.

Table 9 shows the significant predictors of hypogonadism: only SSPG exhibited an AUROC >0.7 (0.707); waist, age, and waist/height ratio exhibited AUROCs between >0.6 and <0.7. The discrimination power of waist circumference, age, and waist/height ratio were mediocre. Again, waist/height ratio was no better than waist circumference alone. Age exhibited an AUROC of only 0.606. The cut-offs for these predictors were: SSPG >224 mg/dL, waist circumference >110 cm, waist/height ratio >0.655, and age >43 years. The AUROC of BMI as a predictor of HYPOG was 0.599 and its cut-off was >32.4, but the *p*-value (0.08) of its AUROC fell short of statistical significance. The best predictor of hypogonadism was an SSPG >224 mg/dL: in fact, 24 out of 48 (50%) IR subjects with an SSPG >224 were also HYPOG; in contrast, only 13 out of the 93 subjects (14%) with an SSPG ≤224 mg/dL were also HYPOG.

**Table 9:** Significant predictors of hypogonadism.

	**Age (years)**	**Waist/height**	**Waist (cm)**	**SSPG (mg/dL)**
AUROC	0.606	0.637	0.640	0.707
*p*-value	0.038	0.027	<0.009	<0.0002
Cut-off value	>43	>0.655	>110	>224
Sensitivity	0.563	0.289	0.816	0.767
Specificity	0.684	0.932	0.447	0.658
Youden Index	0.247	0.222	0.263	0.425

*SSPG values outperformed (acceptable discrimination) Waist, Waist/Height, and Age (mediocre discrimination) at predicting male hypogonadism*.

To evaluate the usefulness of a WC >110 cm at identifying HYPOG subjects we computed total testosterone values in subjects with a WC ≤110 cm and compared it with the total testosterone values found in subjects with a WC >110 cm: 4.4 ± 0.2 (*n* = 111) vs. 3.6 ± 0.3 ng/mL (*n* = 30; *p* = 0.0151), a significant difference. It must be stressed though that a WC ≤110 cm did not prevent HYPOG: in fact, 25/111 subjects (22.5%) with a WC ≤110 cm were HYPOG while 12/30 subjects (40%) with a WC >110 cm were HYPOG.

It is important to note that WC and waist/height ratio predicted HYPOG at a higher level compared with their corresponding level predicting IR: WC cut-offs were >99 cm for IR and >110 for HYPOG; waist/height cut-offs were >0.575 for IR and >0.655 for HYPOG. Another consideration is the fact that a WC >99 cm was better at predicting IR than a WC >110 cm was at predicting HYPOG.

## Discussion

We had to take 3 specific decisions to carry out this research: (a) how to label a subject as IR was the first task. We decided to label a subject as IR when his SSPG value was ≥150 mg/dl since this cut-off has been selected by Reaven's group through the prospective monitoring of non-obese, apparently healthy subjects who, in time, developed clinical syndromes related to IR and aging (20–22 and 24–26); (b) the second task was to decide when to label a subject as HYPOG. We chose a total testosterone level of ≤3 ng/mL as a cut-off value to categorize a subject as HYPOG. This level was selected based on published data, such as the Telecom Study (2), in which—in a population of 1,718 adult male subjects—the fifth percentile of total testosterone was 3.4 ng/mL. On the other hand, the 2nd Annual Andropause Consensus Meeting used a cutoff level of 3.25 ng/mL (27) to categorize subjects as HYPOG. Finally, the Clinical Practice Guideline on Testosterone Therapy in Adult Men with Androgen Deficiency Syndromes (28) suggests a total testosterone cutoff value <3 ng/mL to label males as HYPOG; (c) the third task was to define how to measure WC. WC measurement sounds like an easy measurement to obtain but it is not. The selection of the type of WC to measure is complicated by the fact that waist lacks a definite anatomical reference and, in fact, at least four different waist circumference measurements are in use (29). Two of them use a borrowed anatomical site, either below the lowest rib or above the iliac crest, while the other two are measured either at midway between the above-mentioned anatomical sites or else, at the narrowest waist. We chose the last alternative, more congruent with the lay concept of “waist” and followed the methodology used by Lean et al. (17). It should be noted that NAHNES III measures WC above the iliac crest.

With these criteria, we proceeded to categorize each subject as IR (SSPG ≥150 mg/dL) or NIR (SSPG <150 mg/dL) and as HYPOG (total testosterone ≤3.0 ng/mL) or EUG (total testosterone >3.0 ng/mL).

We studied a masculine cohort with two-thirds of IR population and approximately, one-fourth of HYPOG population. Insulin resistance and hypogonadism were both present in normal weight, overweight and obese subjects in different proportions. A crucial aspect of our study is the fact that the patients were selected on the basis of clinical insulin resistance.

Among our 15 normal weight subjects (all of them highly suspicious of being IR), insulin resistance was present in over half of them, while their ages were not different from the ages of the overweight or obese subjects. Moreover, hypogonadism affected 3 of these 15 normal weight subjects. Two out of three of them were both IR (SSPG 249 and 237 mg/dL) and HYPOG (testosterone 1.5 and 2.7 ng/mL, respectively). Their respective BMIs were 24.3 and 24.6. The other HYPOG subject (testosterone 3.0 ng/mL) with a normal weight (BMI of 23) was NIR (SSPG of 89 mg/dL). The apparently intriguing finding that over a half of the normal-weight subjects were IR underscore the fact that insulin resistance may affect people with a normal BMI (the subjects were indeed clinically selected on the basis of a probable insulin resistance); the fact that 20% of these subjects were also HYPOG might be explained by the fact that 2 out of 3 of them were also IR, underscoring the fact that insulin resistance, by itself or via an associated phenomenon, may induce male hypogonadism, in the absence of obesity.

Among our 66 obese subjects, 9 of them (13.6%) were NIR and 46 patients (69.7%) were EUG. Moreover, 6 of the obese subjects were both NIR and EUG (9.1%). In other words, 6/9 of these obese subjects were spared both from insulin resistance and hypogonadism. These findings support the existence of a subgroup of “metabolically healthy obese patients” and, on the other hand, cast a doubt on the concept that over a half of the obese males are HYPOG.

From our data it is clear that insulin resistance clustered with hypogonadism: in fact, being IR more than doubled the relative risk of being also HYPOG (RR = 2.14), while being HYPOG modestly increased the relative risk of being also IR (RR = 1.305). Moreover, contingency analysis showed that hypogonadism and insulin resistance were significantly interdependent (Chi-square = 4.688, 1 degree of freedom, *p* = 0.0303).

The fact that being HYPOG modestly increases the relative risk of being IR lends credence to the controversial notion that testosterone is an insulin-sensitizing hormone. Moreover, HYPOG subjects exhibited higher levels of SSPG (Table 5), as compared with EUG subjects, further suggesting that testosterone deficiency deteriorates insulin sensitivity. On the other hand, the fact that being IR doubles the relative risk of being HYPOG is more complex to interpret (see ahead the discussion on this issue).

In this investigation, a WC >99 cm emerged as an excellent predictor of insulin resistance. Its AUROC against an SSPG level ≥150 mg/dL was 0.812, which is considered an excellent discrimination. Moreover, in our cohort, 89.74% of the subjects having a WC >99 cm was IR. However, a WC >110 cm was only a mediocre predictor of hypogonadism. In fact, its AUROC against a serum testosterone level ≤3.0 ng/mL was only 0.640, a less than an acceptable discrimination. In contrast, an SSPG ≥224 mg/dl was an acceptable predictor of hypogonadism in our cohort, with an AUROC of 0.707. Moreover, 48 subjects in our cohort had an SSPG >224 mg/dL, and 24 of them (50%) were HYPOG while only 14% of the subjects with an SSPG ≤224 were HYPOG.

Adult males usually tend to gain weight as they age. As this extra fat is localized mainly as visceral fat inside the abdomen their WCs progressively enlarge. This phenomenon is clearly related to the development of insulin resistance. The ATP III criteria for the diagnosis of metabolic syndrome, a clinical surrogate of insulin resistance, demands a WC in males >102 cm, whereas the IDF criteria demands for Europid males a WC >94 cm. The IDF suggests for South American males a WC >90 cm for the diagnosis of metabolic syndrome. In this cohort, a WC >99 cm was the best predictor of an elevated SSPG value (≥150 mg/dL), a value closer to the one suggested by the ATP III criteria (>102 cm) and clearly higher than the value suggested by the IDF for the region (>90 cm). We found that the discrimination ability of a WC >99 cm in predicting IR was excellent.

As WC continues to enlarge with age it may become associated with male hypogonadism. The necessary cut-off for the WC to predict hypogonadism was >110 cm in this cohort (11 cm larger than the cut-off predicting IR) but its discrimination ability in predicting testicular malfunction was only mediocre with an AUROC of only 0.640. Moreover, 22.5% of the subjects with a WC ≤110 cm was labeled as HYPOG and only 11/37 (29.7%) of the HYPOG subjects had a WC >110 cm.

We suspect that neither central obesity nor insulin resistance is directly related to male hypogonadism since there is no described mechanism for them in eliciting this problem. It is conceivable that another associated mechanism is inducing testicular malfunctioning. Theoretically speaking, our prime suspect is obesity-driven hyperleptinemia, since this condition has been reported to disrupt LH signaling on Leydig cells (15). Leptin has, in fact, emerged as the best endocrine predictor of reduced androgens in male obesity. Since we did not measure leptin in this cohort we can only speculate on the matter. Future research should investigate the intriguing relation between hyperleptinemia and male hypogonadism. Should hyperleptinemia eventually emerge as the main driving force of obesity-associated male hypogonadism, then future leptin sensitizers (currently being developed) might solve the obesity-induced testicular malfunction before weight loss occurs.

Our results in normal weight subjects, all of them with a WC ≤99 cm, demonstrate that a normal WC did not protect them from either insulin resistance or hypogonadism. Moreover, 23/94 IR subjects (24.5%) had a WC ≤99 cm. For us, the main lesson to be learned in this regard is that abdominal obesity should not be considered a sine qua non feature for the suspicion of IR. Our results suggest that the ATP III criteria for the diagnosis of metabolic syndrome (a clinical surrogate of insulin resistance) are more applicable to our population than those of the IDF because, (a) abdominal obesity is not an obligated requisite for the diagnosis of metabolic syndrome when using the ATP III criteria while it is a mandatory requirement when the IDF criteria are applied; (b) in this cohort, insulin resistance was present in 8/15 subjects without abdominal obesity and, (c) the WC cut-off to suspect insulin resistance was >99 cm, a figure closer to the 102 cm of the ATP III criteria than to the figure >90 cm suggested by the IDF criteria for South American males.

In summary, we derive one general conclusion and four specific conclusions from this research. The general conclusion is that abdominal obesity in males seems to be associated with two hits on male health: the initial one is the induction of insulin resistance and the second hit, usually years later, is the induction of hypogonadism.

The first specific conclusion is that a WC >99 cm is an excellent predictor of insulin resistance;

The second specific conclusion is that, despite its excellent ability to predict insulin resistance, a WC >99 cm should not be considered a mandatory requirement to suspect its presence. In fact, abdominal obesity (defined by a WC >99 cm) was present in only about three quarters (71/94) or the IR subjects;

The third specific conclusion is that, while a WC >110 cm is only a mediocre predictor of male hypogonadism, the best predictor of it was an SSPG value >224 mg/dL. The relative risk of being HYPOG with an SSPG >224 mg/dL, compared with an SSPG ≤224 mg/dL was 3.58 (50/14);

The fourth, specific conclusion is that a WC >110 cm should not be regarded as an obligated requirement to suspect hypogonadism in obese males. In fact, most HYPOG males (26/37 = 70.3%) had a WC ≤110 cm. Moreover, 23.4% of the subjects with a WC ≤110 cm were HYPOG.

## Ethics statement

This study was carried out in accordance with the recommendations of the Ethics Committee of the Human Reproductive Research Institute with written informed consent from all subjects. All subjects gave written informed consent in accordance with the Declaration of Helsinki. The protocol was approved by the Ethics Committee of the Human Reproductive Research Institute.

## Author contributions

PC: study design, clinical work with patients, data analysis, paper writing. FS: literature review, final paper's modifications to fit submission requirements. AS: PST supervision, laboratory work, data keeping. PV: study design, clinical work with patients, paper review.

### Conflict of interest statement

The authors declare that the research was conducted in the absence of any commercial or financial relationships that could be construed as a potential conflict of interest.

## References

[1] Zumoff B Strain GW Miller LK Rosner W Senie R Seres DS. Plasma free and non-sex-hormone-binding-globulin-bound testosterone are decreased in obese men in proportion to their degree of obesity. J Clin Endocrinol Metab. (1990) 71:929–31. 10.1210/jcem-71-4-929 2401718

[2] Simon D Preziosi P Barrett-Connor E Roger M Saint-Paul M Nahoul K. Interrelation between plasma testosterone and plasma insulin in healthy adult men: the Telecom Study. Diabetologia (1992) 35:173–7. 10.1007/BF00402551 1547923

[3] Haffner SM Karhapaa P Mykkanen L Laakso M. Insulin resistance, body fat distribution, and sex hormones in men. Diabetes (1994) 43:212–9. 10.2337/diab.43.2.212 8288045

[4] Haffner SM Shaten J Stern MP Smith GD Kuller L. Low levels of sex hormone-binding globulin and testosterone predict the development of non-insulin-dependent diabetes mellitus in men. MRFIT Research Group. Multiple Risk Factor Intervention Trial. Am J Epidemiol. (1996) 143:889–97. 10.1093/oxfordjournals.aje.a008832 8610702

[5] Pitteloud N Hardin M Dwyer AA Valassis E Yialamas M Elahi D. Increasing insulin resistance is associated with a decrease in Leydig cell testosterone secretion in men. J Clin Endocrinol Metab. (2005) 90:2636–41. 10.1210/jc.2004-2190 15713702

[6] Pitteloud N Mootha VK Dwyer AA Hardin M Lee H Eriksson KF. Relationship between testosterone levels, insulin sensitivity, and mitochondrial function in men. Diabetes Care (2005) 28:1636–42. 10.2337/diacare.28.7.1636 15983313

[7] Kapoor D Goodwin E Channer KS Jones TH. Testosterone replacement therapy improves insulin resistance, glycaemic control, visceral adiposity and hypercholesterolaemia in hypogonadal men with type 2 diabetes. Eur J Endocrinol. (2006) 154:899–906. 10.1530/eje.1.02166 16728551

[8] Dhindsa S Prabhakar S Sethi M Bandyopadhyay A Chaudhuri A Dandona P. Frequent occurrence of hypogonadotropic hypogonadism in type 2 diabetes. J Clin Endocrinol Metab. (2004) 89:5462–8. 15531498 10.1210/jc.2004-0804

[9] Mulligan T Frick MF Zuraw QC Stemhagen A McWhirter C. Prevalence of hypogonadism in males aged at least 45 years: the HIM study. Int J Clin Pract. (2006) 60:762–9. 10.1111/j.1742-1241.2006.00992.x 16846397 PMC1569444

[10] Mamtani M Kulkarni H Dyer TD Almasy L Mahaney MC Duggirala R. Waist circumference independently associates with the risk of insulin resistance and type 2 diabetes in Mexican American families. PLoS ONE (2013) 8:e59153. 10.1371/journal.pone.0059153 23536864 PMC3594157

[11] Corona G Rastrelli G Monami M Saad F Luconi M Lucchese M. Body weight loss reverts obesity-associated hypogonadotropic hypogonadism: a systematic review and meta-analysis. Eur J Endocrinol. (2013) 168:829–43. 10.1530/EJE-12-0955 23482592

[12] Saad F Yassin A Doros G Haider A. Effects of long-term treatment with testosterone on weight and waist size in 411 hypogonadal men with obesity classes I-III: observational data from two registry studies. Int J Obes. (2016) 40:162–70. 10.1038/ijo.2015.139 26219417 PMC4722240

[13] Dhinsa S Ghanim H Batra M Kuhadiya ND Abuaysheh S Sandhu S. Insulin resistance and inflammation in hypogonadotropic hypogonadism and their reduction after testosterone replacement in men with type 2 diabetes. Diabetes Care (2016) 39:82–91. 10.2337/dc15-1518 26622051 PMC4686848

[14] Magnussen LV Glintborg D Hermann P Hougaard DM Hojlund K Andersen M. Effect of testosterone on insulin sensitivity, oxidative metabolism and body composition in aging men with type 2 diabetes on metformin monotherapy. Diabetes Obes Metab. (2016) 18:980–9. 10.1111/dom.12701 27265844

[15] Huang G Pencina KM Li Z Basaria S Bhasin S Travison TG. Long-term testosterone administration on insulin sensitivity in older men with low or low-normal testosterone levels. J Clin Endocrinol Metab. (2018) 103:1678–85. 10.1210/jc.2017-02545 29373734 PMC6276701

[16] Isidori AM Caprio M Strollo F Moretti C Frajese G Isidori A. Leptin and androgens in male obesity: evidence for leptin contribution to reduced androgen levels. J Clin Endocrinol Metab. (1999) 84:3673–80. 10.1210/jc.84.10.3673 10523013

[17] Lean ME Han TS Morrison CE. Waist circumference as a measure for indicating need for weight management. BMJ (1995) 311:158–61. 10.1136/bmj.311.6998.158 7613427 PMC2550221

[18] Södergard R Backstrom T Shanbhag V Carstensen H. Calculation of free and bound fractions of testosterone and estradiol-17 beta to human plasma proteins at body temperature. J Steroid Biochem. (1982) 16:801–10. 7202083 10.1016/0022-4731(82)90038-3

[19] Vermeulen A Verdonck L Kaufman JM. A critical evaluation of simple methods for the estimation of free testosterone in serum. J Clin Endocrinol Metab. (1999) 84:3666–72. 10.1210/jcem.84.10.6079 10523012

[20] Shen SW Reaven GM Farquhar JW. Comparison of impedance to insulin-mediated glucose uptake in normal subjects and in subjects with latent diabetes. J Clin Invest. (1970) 49:2151–60. 10.1172/JCI106433 5480843 PMC322715

[21] Harano Y Ohgaku S Hidaka H Haneda K Kikkawa R Shigeta Y. Glucose, insulin and somatostatin infusion for the determination of insulin sensitivity. J Clin Endocrinol Metab. (1977) 45:1124–7. 10.1210/jcem-45-5-1124 925138

[22] Pei D Jones CN Bhargava R Chen YD Reaven GM. Evaluation of octreotide to assess insulin-mediated glucose disposal by the insulin suppression test. Diabetologia (1994) 37:843–5. 10.1007/BF00404344 7988789

[23] Hosmer DW Lemeshow S Applied Logistic Regression. 2nd ed New York, NY; Chichester: Wiley (2000).

[24] Yip J Facchini FS Reaven GM. Resistance to insulin-mediated glucose disposal as a predictor of cardiovascular disease. J Clin Endocrinol Metab. (1998) 83:2773–6. 10.1210/jcem.83.8.5005 9709945

[25] Kim MK Reaven GM Chen YD Kim E Kim SH. Hyperinsulinemia in individuals with obesity: role of insulin clearance. Obesity (2015) 23:2430–4. 10.1002/oby.21256 26524351 PMC4701635

[26] Facchini FS Hua N Abbasi F Reaven GM. Insulin resistance as a predictor of age-related diseases. J Clin Endocrinol Metab. (2001) 86:3574–8. 10.1210/jcem.86.8.7763 11502781

[27] Clinical, Affairs Committee of the Endocrine Society Summary from the Second Annual Andropause Consensus Meeting. Available online at: http://www.endo-societyorg/publicpolicy/legislative/upload/andro_cc_summarypdf.2001

[28] Bhasin S Cunningham GR Hayes FJ Matsumoto AM Snyder PJ Swerdloff RS. Testosterone therapy in men with androgen deficiency syndromes: an Endocrine Society clinical practice guideline. J Clin Endocrinol Metab. (2010) 95:2536–59. 10.1210/jc.2009-2354 20525905

[29] Wang J Thornton JC Bari S Williamson B Gallagher D Heymsfield SB. Comparisons of waist circumferences measured at 4 sites. Am J Clin Nutr. (2003) 77:379–84. 10.1093/ajcn/77.2.379 12540397

